# Evaluation of Plasma E-Selectin Concentration as a Risk Marker for Atherosclerotic Vascular Damage in Patients with Early CAD

**DOI:** 10.3390/biom15010022

**Published:** 2024-12-27

**Authors:** Monika Rac, Michal Rac, Andrzej Krzystolik, Krzysztof Safranow, Dariusz Chlubek, Violetta Dziedziejko

**Affiliations:** 1 Department of Biochemistry, Pomeranian Medical University, Powstańców Wielkopolskich 72, 70-111 Szczecin, Poland; chrissaf@mp.pl (K.S.); dchlubek@pum.edu.pl (D.C.); viola@pum.edu.pl (V.D.); 2 Department of Diagnostic Imaging and Interventional Radiology, Pomeranian Medical University, Unii Lubelskiej 1, 71-252 Szczecin, Poland; hno15hno@gmail.com; 3 Department of Cardiology, County Hospital in Szczecin, Arkońska 4, 71-455 Szczecin, Poland; akrzystolik@poczta.onet.pl

**Keywords:** E-selectin, atherosclerosis, triglycerides, CAD risk marker

## Abstract

Background: Inflammation markers in the blood may indicate a higher risk of unstable atherosclerosis. Selectins, a group of transmembrane glycoproteins, contribute to inflammation by helping certain blood cells bind to the endothelium. Methods: The study included 100 patients with stable early-onset coronary artery disease (CAD), 75 men (aged 50–54) and 25 women (aged 55–64). Tests performed included biochemical analysis, ultrasound, and Doppler imaging of arteries and peripheral vessels. A biochemical control group of 50 cases without CAD (74% men, average age 48 ± 3.20 years) was also studied. Results: Higher triglyceride levels were strongly linked to elevated plasma E-selectin levels. However, no significant relationship was found between plasma E-selectin levels and biochemical, clinical, radiographic, or echographic measures. Conclusion: Plasma E-selectin levels are not a reliable marker for detecting atherosclerotic plaques or related problems in individuals with stable, well-managed CAD. While E-selectin levels can be measured in clinical labs using immunoassays, they cannot replace standard cardiological and vascular imaging tests for diagnosing cardiac or vascular conditions.

## 1. Introduction

Atherosclerosis involves both structural and functional impairment of the vascular endothelium. Inflammation is recognized as a significant factor in atherogenesis and the progression of coronary artery disease (CAD). Circulating markers of inflammation are recognized as risk factors for unstable atherosclerosis [1]. It takes five steps to start the inflammatory response: adhesion (which involves integrins), activation (which involves cytokines), diapedesis (where leukocytes cross the endothelial barrier to reach the antigen site, helped by rising levels of chemokines), and margination (where leukocytes move from the bloodstream to the vessel wall) [2,3].

**Figure 1:**
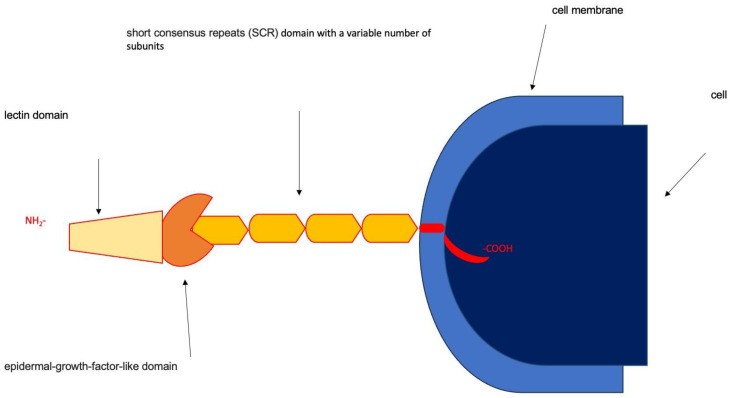
Selectin structure diagram.

Selectins are transmembrane glycoproteins that play a crucial role in the inflammatory response. The function of these proteins is to regulate the adhesion of certain blood cells to the endothelium. They assist leukocytes and platelets in moving away from the centre of the bloodstream by enabling them to roll along the surface of the vascular endothelium [4]. Selectins exhibit structural similarities. Figure 1 depicts the structure of selectins. Each molecule consists of an N-terminal external lectin domain (homologous to calcium-dependent lectins), followed by a homologous to epidermal growth factor domain (EGF) and, in variable numbers (2–9) depending on the selectin, homologous domains to complement regulatory proteins and a transmembrane fragment that binds the whole molecule to the cell membrane, and finally, a short C-terminal intracellular fragment [5]. Monocytes, lymphocytes, and granulocytes express L (leukocyte) selectin on their surfaces. This promotes connections between lymphocytes and endothelial cells in lymph nodes. Ligands for it are mucosal addressin cell adhesion molecule 1 (MAdCAM-1), gly-cosylation-dependent cell adhesion molecule 1 (GlyCAM-1), and receptor CD34. P (platelet) selectin is found in the alpha granules of platelets and in the Weibel–Pallade bodies of endothelial cells. The ligands are P-selectin glycoprotein ligand-1 (PSGL-1) and receptor CD24. Endothelial cells generate and store selectin E, also known as ELAM-1. Pro-inflammatory cytokines only activate it on the endothelium, causing rolling endothelial cells to move slowly [6]. Inflammatory cytokines such as IL-1, TNF, interferon, and bacterial lipopolysaccharides lead to increased expression of the E-selectin gene. Platelet factor 4 (PF4), released by active platelets, acts as a ligand for the LRP receptor (also called receptor for apo E). Nuclear factor kappa-light-chain-enhancer of activated B cells (NF-κB) is turned on when ligands bind to them, and endothelial cells make more mRNA for E-selectin [7]. Hypoxia promotes surface expression, but hypothermia inhibits it [8]. E-selectin ligands are PSGL-1 and ESL-1. Both proteins have not yet been fully defined. The soluble form of E-selectin circulates in plasma. Higher levels of this protein are related to systemic inflammation, activation, and endothelial cell damage. The soluble part of E-selectin is higher in people who had allergic reactions, septic shock, inflammatory vascular disease, post-transplant graft-versus-recipient disease, high blood pressure, diabetes, and high cholesterol. However, its association with atherosclerotic disease and significance as a prognostic indicator are debatable [9,10].

CAD is a multifactorial disease resulting from the interaction of genetic and traditional risk factors. Half of the CAD GWAS loci do not associate with traditional risk factors. Hypercholesterolemia induces atherosclerosis and is a risk factor for CAD through increases in atherogenic biomolecules, including proinflammatory cytokines as interleukins (IL-1, IL-2, IL-6, IL-8), tumor necrosis factor-alpha (TNF-α)], expression of intercellular adhesion molecule-1 (ICAM-1), vascular cell adhesion molecule-1 (VCAM-1), E-selectin, and CRP protein [11,12]. Vascular endothelial cells have critical roles in atherosclerosis and after activation, they express adhesion molecules necessary for monocyte rolling and attachment (e.g., E-selectin, ICAM1 and VCAM1). Weakening of their cell–cell junctions can facilitate monocyte transmigration into the intima [13]. The genetic variation in E-selectin and some polymorphisms in gene coding this protein are significantly linked to stable angina and myocardial infarction [14]. In fact, in patients with congenital heart disease, E-selectin levels reflect the severity of hypoxia [15]. Furthermore, Safonova et al. [16] reports E-selectin as an endothelial dysfunction marker in CAD patients. In that study, E-selectin level in plasma decreased from 57.25 to 46.05 ng/mL after 12-month therapy with perindopril. It is generally accepted that endothelial dysfunction plays a critical role in the progression of atherosclerotic lesions. One marker of endothelial dysfunction is E-selectin. Researchers believe that E-selectin, a biomarker of endothelial activation, contributes to the development of subclinical heart failure (HF) [17] and serves as a biomarker of chronic inflammation in the biopsies of individuals with HF due to dilated cardiomyopathy [18]. Patients with documented CAD showed a significant increase in plasma E-selectin levels compared to those with normal coronary arteries or healthy controls [19], even when they exercised [20]. Plasma E-selectin levels were significantly higher in the acute myocardial infarction and unstable angina groups than in the stable angina and control groups [21]. This protein was also significantly higher in patients with acute coronary syndrome compared with CAD patients and healthy controls [22]. Patel et al. demonstrated that elevated levels of E-selectin in young adulthood are linked to poorer left ventricular (LV) systolic function during midlife among African Americans [23]. E-selectin mediates adhesion and transmigration of leukocytes to the vascular endothelial wall and thus may promote plaque growth and instability. A prospective study of patients with documented CAD revealed higher levels of E-selectin in those who would die from cardiovascular causes in the future [24].

Molecular studies showed that single nucleotide polymorphisms (SNPs) in the E-selectin gene are associated with an increased risk of developing subclinical atherosclerosis in a group of Mexican individuals [25], and are associated with ischemic heart disease in Iraqi [14] and Iranian [26] patients. A study from Poland [27] reported that two variations in the E-selectin gene combined with high cholesterol make people much more likely to develop CAD. Others [28] say that SNPs in the E-selectin gene are genetic factors that change the risk of myocardial attack. In Chinese patients, a mutation in the E-selectin gene at codon 128 has been linked to an elevated risk of CAD, with an odds ratio of 2.21 (95% CI: 1.20–4.07) [29]. Additionally, lowering the expression of the LDLR or CD36 receptor genes also helped monocytes stick to endothelial cells and move across them by raising the expression of adhesion molecules like VCAM-1 and ICAM-1, E-selectin, and P-selectin, thus promoting the initiation of atherosclerosis [30].

On the other hand, researchers observed no significant differences in E-selectin levels between patients with high- and low-risk coronary artery disease (CAD) [31]. Galvani et al. discovered that E-selectin levels were higher when clinically significant atherosclerosis was present, but they did not increase during the unstable phase of the disease. The authors concluded that E-selectin is not a reliable indicator of atherosclerotic plaque [32]. There were no significant differences in E-selectin levels between CAD patients and healthy controls in the Chinese population in Singapore [33]. A different study found that soluble E-selectin levels did not significantly decrease after statin treatment in the CAD group when compared to baseline [34]. Soluble E-selectin measurement was not a key biomarker for calcific aortic stenosis in patients with significant CAD [35] or a predictor of atherosclerosis presence or severity in those with familial hypercholesterolaemia [36]. The parasympathetic nervous system is actively involved in the monitoring and modulation of inflammatory processes, but parasympathetic modulation of inflammation via the vagus nerve may influence certain inflammatory molecules more than others. Alen et al. used a large, nationally representative sample (N = 836) from the United States and found no significant link between heart rate variability (HRV), a measure of parasympathetic activity, and inflammatory adhesion molecules like E-selectin-3 in the blood [37].

Different studies sometimes show conflicting results. Therefore, the search for biochemical markers of early CAD remains valid. The involvement of inflammatory parameters in the pathogenesis of CAD has been intensively studied in recent years. In previous studies, we have investigated the utility of various parameters, including IL6, VEGF, and TNF, as markers of CAD [38,39]. We have shown very weak correlations of plasma VEGF and TNF levels with biochemical cardiovascular risk factors [40]. On the other hand, serum IL-6 levels are clearly associated with hsCRP levels in patients with early-onset CAD [41]. Both proteins are biomarkers of inflammation. In the present study, we investigated the association between plasma E-selectin levels and the presence of known biochemical and clinical risk factors for cardiovascular disease, together with cardiac morphology and radiological parameters of atherosclerotic progression in patients with early-onset CAD. Understanding this group of patients is essential for preventive cardiology. Immunoassays are precise, reliable, and ready for use in hospital labs. We examined whether measuring this pro-inflammatory protein in plasma could be an alternative to traditional methods for assessing vascular status, like echocardiography and vascular Doppler. To our best knowledge, in the available literature there are no studies on the associations of E-selectin with so many cardiac and vascular parameters in stable CAD patients.

This study aimed to answer two questions. Firstly, Is plasma E-selectin a biomarker for atherosclerotic progression in patients with CAD? Secondly, can E-selectin measurement replace the traditional cardiac or radiological assessments of vascular status?

## 2. Materials and Methods

### 2.1. Study Groups

The study included 100 individuals with early-onset coronary artery disease, consisting of 75 men aged 50 and younger and 25 women aged 55 and younger. All patients demonstrated clinical stability. The diagnostic criteria for CAD aligned with those previously established in our research [42]. Patients were excluded from the study if they had hemodynamically significant congenital or acquired heart disease that could affect the cardiac picture (e.g., cardiomyopathy, pericarditis, endocarditis). Patients with acute coronary syndrome and symptomatic heart failure (NYHA ≥ 2) were also excluded from the study. Additional exclusion criteria were type 1 diabetes, renal impairment (serum creatinine > 3 mg/dL), severe liver failure, thyroid illness, and active immunological or malignant diseases. Table 1 shows the characteristics of the CAD patients in detail. A biochemical control group of 50 non-CAD individuals (74% male, mean age 48 ± 3.20 years) was included, with data shown in Table 2. The study followed the Declaration of Helsinki and ethical approval was obtained from the ethics council at Pomeranian Medical University (No. BN-001/162/04). Every participant provided informed consent before participating in the study.

### 2.2. Clinical Data Collection

All patients had their height, weight, waist and hip circumference, and blood pressure measured. The body mass index (BMI), waist-to-hip ratio (WHR), and mean arterial pressure (MAP) were calculated for each patient.

They underwent biochemical tests (using a Cobas 6000 analyser from Roche, Warsaw, Poland) and echocardiographic evaluations (using a Medison SA 9900 machine, Samsung, Daegu, Republic of Korea). Additionally, Doppler imaging (using a USG Doppler machine, Technos Easaote, Pulawy, Poland) of the carotid and peripheral arteries was conducted. Echocardiography encompassed a standard evaluation of anatomical parameters, left ventricular diastolic function (LVDF), left ventricular ejection fraction as per Simpson [43], left ventricular mass according to the Devereux equation [44], and left ventricular mass index as per Simone [45]. Doppler ultrasound was used to assess the thickness of atherosclerotic plaque at the carotid bifurcation (common carotid artery, CCA). It also measured the density and thickness of the intima-media complex (IMC) in the CCA and the brachial artery. The ankle-brachial index (ABI) test was calculated. In our previous publication, we clearly explained the technique for sampling, storing, and quantifying E-selectin using ELISA methods [38]. Plasma concentrations of E-Selectin were measured using commercially available enzyme-linked immunosorbent assay (ELISA) Quantikine kits (cat. No. DSLE00; R&D Systems, Bio-Techne Ltd.; 19 Barton Lane, Abingdon Science Park, Abingdon, UK) according to the manufacturer’s protocol. Before the assay, the samples were diluted 10× with sample diluent. The detection limit of the ELISA for E-selectin was 0.009 ng/mL. The intra-assay precision (CV%) was 5.1–6.9% and the inter-assay precision was 7.3–8.6%. Absorbance was read at 450 nm using automated Microplate Reader ELX 808IU (Bio-Tek Instruments Inc., Winooski, VT, USA). The results were analyzed using a quadratic curve fit. The calibration was performed with recombinant human E-Selectin in concentration range 0.125–8 ng/mL.

### 2.3. Statistical Analysis

In most cases, the parameter distributions of the quantitative clinical characteristics were significantly different from normal distribution (Shapiro–Wilk test), which is common in biomarker studies. Therefore, non-parametric Mann–Whitney U test and Spearman’s rank correlation coefficient (Rs) were used in the statistical calculations. Multiple linear regression analysis with parameter distributions normalized when needed via log-transformation was performed to find independent predictors of plasma E-selectin concentration.

Due to the large number of parameters tested, a very large number of statistical tests (154 comparisons, correlations, or regression analyses in total) were performed. To account for the effect of multiple testing on false positives, the classic Bonferroni correction was applied by dividing standard significance level 0.05 by the number of tests, resulting in a Bonferroni-corrected significance level of *p* = 0.05/154 = 0.00032.

## 3. Results

Comparing the morphology results of the two groups (Table 1 and Table 2) and considering the Bonferroni correction, the statistically significant differences in these parameters are between RBC (*p* = 0.000205), hemoglobin (*p* = 0.000001), and hematocrit (*p* = 0.000011). The two groups also differed significantly in lipidogram results after Bonferroni correction, i.e., total cholesterol (*p* = 0.000002), HDL cholesterol (*p* = 0.000001), and apoA1 (*p* = 0.000012). Plasma concentrations of E-selectin (Table 3) and cytokines were not statistically significantly different between groups. Male gender is a known predisposing factor for coronary artery disease, which is reflected in the high proportion of men among the patients studied. Therefore, to verify the possible influence of the patient’s sex on the results obtained, an additional analysis was performed dividing the CAD patients into male and female subgroups. However, in the CAD group, E-selectin concentrations (ng/mL) were higher in the male group (38.11 ± 1.48 ng/mL) than in the female group (31.22 ± 2.01 ng/mL), but the statistical significance of this association (*p* = 0.011) does not pass the Bonferroni correction. This suggests that the observed correlation across the study group was not influenced by gender.

**Figure 2:**
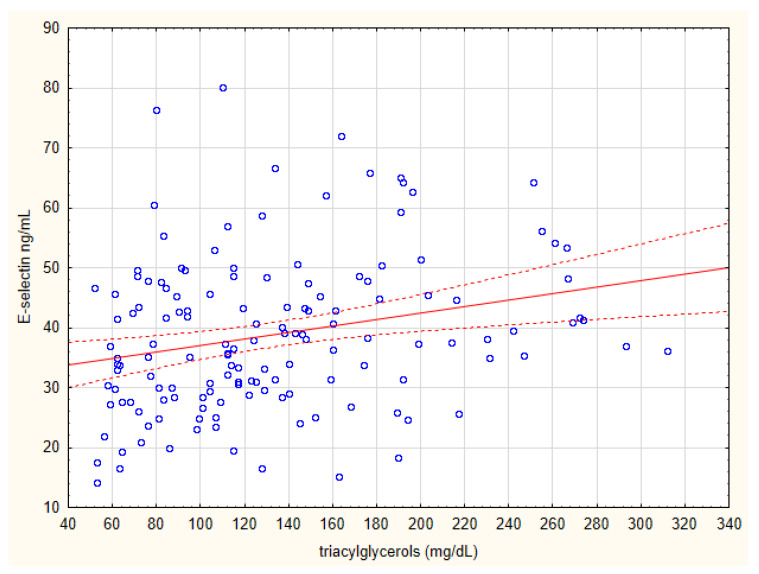
Scatterplot of E-selectin concentration and triglyceride concentration.

**Figure 3:**
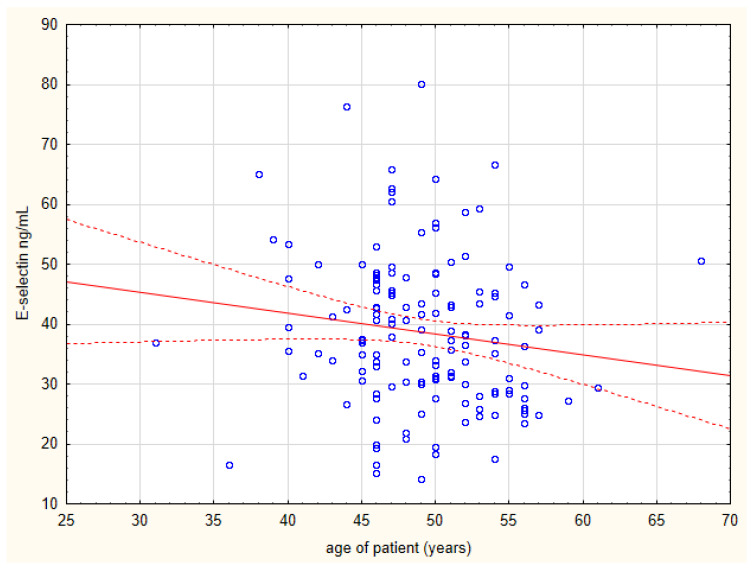
Scatterplot of E-selectin concentration and patient age.

Table 4 presents laboratory parameters associated with E-selectin concentration in any of two groups, applying standard significance criterion of *p* = 0.05. When Bonferroni correction is applied, the positive correlation between E-selectin concentration and triglyceride concentration remains statistically significant in the CAD group, as does the correlation between E-selectin concentration and hemoglobin and hematocrit in the control group. Table 5 shows clinical parameters associated with E-selectin concentration in the CAD group, applying standard significance criterion of *p* = 0.05. None of these associations survived the Bonferroni correction. Scatterplots of E-selectin concentration versus triglyceride concentration and patient age are shown in Figure 2 and Figure 3. The other quantitative biochemical, clinical, radiological, and echographic parameters that were looked at in the CAD group (Table 1) did not show statistically significant associations with plasma E-selectin concentration, even at *p* = 0.05 level. However, we observed a positive association between E-selectin concentration and treatment with the anticoagulant acenocoumarol (*p* = 0.035), but it lost significance after Bonferroni correction. Triglyceride concentration and age of patient as predictors of plasma E-selectin levels were calculated. Linear regression analysis indicated that elevated triglyceride concentration was a significant independent predictor of increased plasma E-selectin levels (Table 6).

## 4. Discussion

In our study, plasma E-selectin concentrations determined via ELISA did not differ in the concentration range (ng/mL) from those obtained by other investigators [46,47,48]. Similar to others, we also observed higher E-selectin concentrations in the male subgroup compared to females [49]. The lack of statistically significant differences between the CAD and control groups in our study is also similar to the observations of other authors [50]. However, some investigators observed that the mean serum levels of soluble E-selectin were significantly higher in the CAD group than in the control group [26,51]. Some studies have shown that CAD patients have higher average E-selectin levels compared to controls, which may be linked to active inflammation [52]. Rość et al. [53] found that E-selectin levels in patients with ischemic heart disease planned for CABG were significantly higher in the preoperative period and at the time of extracorporeal circulation initiation than in healthy subjects. Yamada et al. [54] found that E-selectin levels were higher before CABG but lower in patients who received heparin. They linked this decrease to the anticoagulant effects of heparin. Our patients were also treated with anticoagulants and statins and were also included in the study at least 30 days after the acute cardiovascular episode, i.e., at a time when the plasma inflammation coefficients could have decreased significantly. This could be an explanation for the lack of differences in E-selectin levels between the two groups in our study.

High triglyceride levels are a major risk factor for myocardial infarction, even when LDL cholesterol is well-controlled with statins. This residual cardiovascular risk persists despite optimal statin treatment [55]. In our study, we found a positive correlation between E-selectin and triglyceride levels in CAD patients. Triglycerides were also an independent predictor of high E-selectin levels. This likely occurs because triglycerides, being small particles, can penetrate the intima-media complex (IMC) and trigger an inflammatory response in the endothelium. This inflammation could increase E-selectin levels, but there is a lack of in vivo validation in our study. Similar observations have been reported by other authors in a group of young people with type 1 diabetes [56], and in adult dyslipidemic patients [57]. Consistent with results from other studies, we did not find a significant negative correlation between E-selectin and HDL cholesterol [58]. An increase in E-selectin is reported as an early marker of vascular oxidative stress [59]. Reducing cardiovascular risk factors may help lower E-selectin levels [60].

Conger et al. [61] reported a notable increase in adherent leukocytes in pulmonary microvascular endothelial cell cultures influenced by hemoglobin, in comparison to control groups. This might clarify the surprising positive correlation observed between hemoglobin and E-selectin levels in healthy individuals in our study. In patients with congenital heart disease, E-selectin levels correlated positively with red blood cells, hemoglobin concentration, and hematocrit [15,62]. Certain blood flow disorders are associated with cardiovascular disease, atherosclerosis, diabetes, metabolic syndrome, and lipid disorders. We can identify these disorders using plasma protein composition, hematocrit levels, and red blood cell properties [63]. However, these disorders should not be present in healthy individuals. The positive correlation between E-selectin and hemoglobin, as well as hematocrit, in healthy subjects is probably coincidental.

Additionally, an intriguing finding in our study is the negative, though borderline significant, relationship between E-selectin levels and age in CAD patients. This result was not noticed in the control group (*p* = 0.32), and we found no similar observations in the literature. The term “inflammaging” describes how atherosclerotic lesions worsen with age. Our result might reflect stronger inflammatory responses in younger patients or could simply be a random finding. There is lack of in vivo validation for this observation in our study.

In our study, we found a positive correlation between plasma E-selectin concentration and right ventricular end-diastolic diameter. However, this correlation did not pass the Bonferroni correction. Other authors have also reported no consistent associations between concentrations of E-selectin and other proinflammatory markers and echocardiographic measurements [64,65]. However, increased expression of adhesion molecules, including E-selectin, on the endothelium of atrial, valvular, and myocardial blood vessels has been described [66]. In the cited study, E-selectin expression was significantly positively correlated with left atrial and left ventricular diameter and right atrial pressure. The prevalence of right ventricular dysfunction is known to increase with the severity of left ventricular failure. Right ventricular dysfunction is associated with increased risk and mortality in post-MI patients [67]. These findings need further validation.

The association between plasma E-selectin levels and plaque length did not meet the Bonferroni correction. Other studies show that plaque size is not the main factor in the risk of acute coronary syndrome [68]. The key factor is plaque progression, which involves an increase in fibro-fatty material relative to fibrous material [69]. Some research suggests that levels of adhesion molecules, including E-selectin, may be higher in patients with low-grade vascular lesions [4]. These molecules are known to rise in cases of endothelial activation and may help identify patients with unstable carotid plaques [70]. However, measuring E-selectin levels does not appear to be a good marker for patients with stable, treated CAD.

Meta-analysis is an ideal tool to identify the real association while removing the heterogeneity between studies. This research showed that some of the E-selectin polymorphisms were associated with an increased risk of CAD and vascular diseases [71,72]. Other meta-analyses also showed that statins significantly reduced E-selectin, independently of baseline lipid profile and other study and patient characteristics [73]. The trials did not show whether the reduction in selectin mediated the reduction in cardiovascular risk with these agents. The researchers noted [74] that the sensitivity of E-selectin as a diagnostic or prognostic indicator may vary with different diseases and their progression. They recommend further research to establish standardized thresholds and to fully understand the pathophysiological role of E-selectin. The additional testing of E-selectin in stable CAD patients could address previous research questions.

Our study has some limitations. First, we included a control group of healthy individuals without a history of coronary artery disease. Including an additional cohort of individuals with CAD during an acute cardiovascular episode would be most appropriate. Second, we measured only plasma levels of one selectin: E- selectin. Measuring L-selectin and P-selectin with E-selectin could provide a more comprehensive understanding of the findings. Third, while the group sizes seem adequate, larger groups might help resolve results that are only borderline significant. Lastly, concentrating solely on stable CAD patients limits the study’s scope and clinical relevance.

## 5. Conclusions

### 5.1. Is There an Association Between Plasma E-Selectin Levels and Stable CAD or the Presence of Known Biochemical and Clinical Risk Factors for Cardiovascular Disease?

Although E-selectin is recognised as a suitable plasma marker of inflammation and endothelial dysfunction [62], its plasma concentration cannot serve as a marker of atherosclerotic lesions and associated dysfunction in patients with stable and treated CAD. Its positive association with triacylglycerol plasma concentration in patients with stable CAD needs further investigation.

### 5.2. Could Plasma Measurement of E-Selectin Replace Classical Cardiac or Radiological Examination of Vascular Status?

In the present study, we demonstrated the lack of a clear association of E-selectin concentrations with analyzed cardiovascular and radiological vascular parameters. Determination of plasma E-selectin levels cannot be a surrogate test for coronary and peripheral vascular status and cannot replace classical cardiological and radiological vascular examination.

Future research should include patients with unstable CAD and examine the role of additional inflammatory markers. Next research should focus on clarifying the mechanisms regulating E-selectin level and its interaction with other biomarkers to enhance predictive capabilities.

## Figures and Tables

**Table 1:** The clinical, biochemical, echocardiography, and radiological CAD patients’ parameters (number of patients: 100).

Parameter in CAD Group	Value
Past MI	70%
Time since diagnosis of MI to joining the program (years)	3.20 ± 0.74
Age of the first MI (years)	44.0 ± 5.6
Age of patient (years)	49.9 ± 5.91
Gender (% males)	75%
Weight (kg)	83.4 ± 17.0
Waist (cm)	98.3 ± 12.5
BMI (kg/m^2^)	28.1 ± 3.98
WHR	0.96 ± 0.09
Systolic BP (mmHg)	127 ± 14.2
Diastolic BP (mmHg)	77.0 ± 9.01
Heart rate (1/min)	70.7 ± 12.1
MAP (mmHg)	93.8 ± 9.35
History of hypertension	66%
Age at diagnosis of hypertension (years)	42.6 ± 8.6
Cigarette smokers	89%
Years smoking	18.9 ± 9.8
Past CABG	37%
Past PTCA	71%
Diabetes type 2	13%
Glucose (mg/dL)	107 ± 24.8
Statins	96%
Total cholesterol (mg/dL)	173 ± 40.4
LDL-cholesterol (mg/dL)	102 ± 36.2
HDL-cholesterol (mg/dL)	48.4 ± 11.5
ApoA1 (mg/dL)	154 ± 38.4
ApoB/ApoA1	0.53 ± 0.15
Lp(a) (mg/dL)	40.3 ± 49.3
Triacylglycerols (mg/dL)	136 ± 57.1
Anti-platelet drugs (Aspirin)	90%
Beta-blockers	88%
ACEI	80%
Diuretics	31%
Calcium channel blockers	18%
ARB	17%
LVEF (%)	53.6 ± 11.1
LVMI (g/m^2^)	183 ± 62.3
Left ventricular end–diastolic diameter (mm)	51.3 ± 7.17
Left ventricular end–diastolic volume (mL)	121 ± 43.4
Left atrium diameter (mm)	38.6 ± 5.71
LVDF normal	38%
LVDF impaired	54%
LVDF pseudonormal	8%
Right ventricular end–diastolic diameter (mm)	32.9 ± 5.60
Right ventricular mean systolic pressure (mmHg)	22.0 ± 6.27
DT (ms)	221 ± 69.5
E/A ratio	1.12 ± 0.37
TNF (pg/mL)	1.33 ± 0.36
IL-6 (pg/mL)	1.69 ± 2.77
VEGF (pg/mL)	236 ± 17.2
hsCRP (mg/L)	1.82 ± 2.7
Platelets (G/L)	218 ± 44.6
Hemoglobin (g/dL)	14.8 ± 1.14
Hematocrit (%)	43.9 ± 3.17
RBC (T/L)	4.91 ± 0.42
MCV (fL)	89.6 ± 4.40
MPV (fl)	10.6 ± 0.09
WBC (G/L)	6.80 ± 0.22
ABI	1.16 ± 0.03
IMC cca (mm)	0.81 ± 0.01
IMC ba (mm)	0.57 ± 0.01
PLA present (%)	76%
PLA thickness (mm)	1.41 ± 0.08
PLA length (mm)	7.71 ± 0.33
PLA density (AU)	70.0 ± 3.20

MI—myocardial infarction, BMI—body mass index, WHR—waist-to-hip ratio, MAP—mean arterial pressure, CABG—coronary artery bypass grafting, PTCA—percutaneous transluminal coronary angioplasty, ACEI—angiotensin 1 converting enzyme inhibitors, ARB—angiotensin 2 receptor blockers, LVEF—left ventricular ejection fraction, LVMI—left ventricular mass index, LVDF—left ventricle diastolic function, DT—deceleration time, TNF—tumor necrosis factor α, IL6—interleukin 6, VEGF—vascular endothelial growth factor, RBC—red blood cells, MCV—mean corpuscular volume, MPV—mean platelet volume, WBC—white blood cells, ABI—ankle-brachial index, IMC cca—intima-media complex of common carotid arteries, IMC ba—intima-media complex of brachial arteries, PLA—plaque of common carotid arteries and bifurcation.

**Table 2:** The biochemical parameters in the control group (number of patients: 50).

Parameter in Control Group	Value
Age of patient (years)	48 ± 3.20
Gender (% males)	74%
Glucose (mg/dL)	96.0 ± 4.07
Total cholesterol (mg/dL)	217 ± 6.36
LDL-cholesterol (mg/dL)	126 ± 5.74
HDL-cholesterol (mg/dL)	66.0 ± 2.15
ApoA1 (mg/dL)	177 ± 5.16
ApoB/ApoA1	0.51 ± 0.03
Lp(a) (mg/dL)	23.2 ± 9.83
Triacylglycerols (mg/dL)	110 ± 8.52
TNF (pg/mL)	1.51 ± 0.005
IL-6 (pg/mL)	1.47 ± 0.33
VEGF (pg/mL)	220 ± 77.5
hsCRP (mg/L)	2.10 ± 0.26
Platelets (G/L)	223 ± 11.2
Hemoglobin (g/dL)	13.7 ± 0.15
Hematocrit (%)	41.4 ± 0.43
RBC (T/L)	4.64 ± 0.06
MCV (fL)	90.0 ± 0.72
MPV (fL)	10.3 ± 0.14
WBC (G/L)	5.50 ± 0.16

**Table 3:** The E-selectin plasma concentration comparisons between the CAD and control groups, and between male and female subgroups of the CAD group.

E-Selectin	ng/mL	*p*-Value *
CAD group	35.58 ± 1.21	0.11
control group	41.17 ± 1.99	
CAD male subgroup	38.11 ± 1.48	0.011
CAD female subgroup	31.22 ± 2.01	

* Mann–Whitney U test. Bonferroni-corrected significance level *p* = 0.00032.

**Table 4:** The correlations between circulating E-selectin concentration (ng/mL) and laboratory parameters of early-onset CAD patients and of the control group.

Parameter	Correlations for CAD Patients (*n* = 100)		Correlations for Control Group (*n* = 50)	
	Rs	*p*-Value	Rs	*p*-Value
RBC (T/L)	0.19	0.067	0.37	0.0069
Hemoglobin (g/dL)	0.31	0.0027	0.50	**0.00015**
Hematocrit (%)	0.30	0.0040	0.50	**0.00032**
HDL-cholesterol (mg/dL)	−0.24	0.018	−0.28	0.042
Triacylglycerols (mg/dL)	0.38	**0.00018**	0.24	0.093
ApoB/ApoA1	0.26	0.012	-	-
Glucose (mg/dL)	0.10	0.33	0.314	0.025

Rs—Spearman rank correlation coefficients. Associations surviving Bonferroni correction (*p* = 0.00032) are marked bold.

**Table 5:** The correlations between circulating E-selectin concentration (ng/mL) and quantitative clinical parameters of early-onset CAD patients.

Parameter	Correlations for CAD Patients (*n* = 100)	
	Rs	*p*-Value
Age of patient (years)	−0.34	0.00087
Right ventricular end–diastolic diameter (mm)	0.21	0.046
PLA length (mm)	−0.43	0.0064

Rs—Spearman rank correlation coefficients. Bonferroni-corrected significance level *p* = 0.00032.

**Table 6:** The multiple linear regression model with log-transformed plasma E-selectin concentration as the dependent variable. With Bonferroni correction for multiple comparisons 0.0003247 is statistically significant. In fact, the only statistically significant result is one that is lower than this value.

Independent Variables	Standardized β Coefficient (95%CI)	*p*-Value
Triacylglycerols (mg/dL)	+0.37	**0.00025**
Age of patient (years)	−0.22	0.023

Associations surviving Bonferroni correction (*p* = 0.00032) are marked bold.

## Data Availability

The full study records of each patient are kept in the Department of Biochemistry at Pomeranian Medical University.
